# Best practices for fNIRS publications

**DOI:** 10.1117/1.NPh.8.1.012101

**Published:** 2021-01-07

**Authors:** Meryem A. Yücel, Alexander v. Lühmann, Felix Scholkmann, Judit Gervain, Ippeita Dan, Hasan Ayaz, David Boas, Robert J. Cooper, Joseph Culver, Clare E. Elwell, Adam Eggebrecht, Maria A. Franceschini, Christophe Grova, Fumitaka Homae, Frédéric Lesage, Hellmuth Obrig, Ilias Tachtsidis, Sungho Tak, Yunjie Tong, Alessandro Torricelli, Heidrun Wabnitz, Martin Wolf

**Affiliations:** a Boston University, Neurophotonics Center, Biomedical Engineering, Boston, Massachusetts, United States; b Massachusetts General Hospital, Harvard Medical School, MGH/HST Athinoula A. Martinos Center for Biomedical Imaging, Department of Radiology, Charlestown, Massachusetts, United States; c University Hospital Zurich, University of Zurich, Department of Neonatology, Biomedical Optics Research Laboratory, Neonatology Research, Zurich, Switzerland; d University of Bern, Institute for Complementary and Integrative Medicine, Bern, Switzerland; e Université de Paris, CNRS, Integrative Neuroscience and Cognition Center, Paris, France; f Università di Padova, Department of Social and Developmental Psychology, Padua, Italy; g Chuo University, Faculty of Science and Engineering, Applied Cognitive Neuroscience Laboratory, Tokyo, Japan; h Drexel University, School of Biomedical Engineering, Science and Health Systems, Philadelphia, Pennsylvania, United States; i Drexel University, College of Arts and Sciences, Department of Psychology, Philadelphia, Pennsylvania, United States; j Drexel University, Drexel Solutions Institute, Philadelphia, Pennsylvania, United States; k University of Pennsylvania, Department of Family and Community Health, Philadelphia, Pennsylvania, United States; l Children’s Hospital of Philadelphia, Center for Injury Research and Prevention, Philadelphia, Pennsylvania, United States; m University College London, DOT-HUB, Department of Medical Physics and Biomedical Engineering, Biomedical Optics Research Laboratory, London, United Kingdom; n Washington University School of Medicine, Department of Radiology, St. Louis, Missouri, United States; o University College London, Department of Medical Physics and Biomedical Engineering, London, United Kingdom; p Washington University School of Medicine, Mallinckrodt Institute of Radiology, St. Louis, Missouri, United States; q Concordia University, Department of Physics and PERFORM Centre, Multimodal Functional Imaging Lab, Montreal, Québec, Canada; r McGill University, Biomedical Engineering Department, Multimodal Functional Imaging Lab, Montreal, Québec, Canada; s Tokyo Metropolitan University, Department of Language Sciences, Tokyo, Japan; t Polytechnique Montréal, Department Electrical Engineering, Montreal, Canada; u University Hospital Leipzig, Max-Planck-Institute for Human Cognitive and Brain Sciences and Clinic for Cognitive Neurology, Leipzig, Germany; v Korea Basic Science Institute, Research Center for Bioconvergence Analysis, Ochang, Cheongju, Republic of Korea; w Weldon School of Biomedical Engineering Purdue University, West Lafayette, Indiana, United States; x Politecnico di Milano, Dipartimento di Fisica, Milan, Italy; y Consiglio Nazionale delle Ricerche, Istituto di Fotonica e Nanotecnologie, Milan, Italy; z Physikalisch-Technische Bundesanstalt, Berlin, Germany

**Keywords:** functional near-infrared spectroscopy, guidelines, publication best practices

## Abstract

The application of functional near-infrared spectroscopy (fNIRS) in the neurosciences has been expanding over the last 40 years. Today, it is addressing a wide range of applications within different populations and utilizes a great variety of experimental paradigms. With the rapid growth and the diversification of research methods, some inconsistencies are appearing in the way in which methods are presented, which can make the interpretation and replication of studies unnecessarily challenging. The Society for Functional Near-Infrared Spectroscopy has thus been motivated to organize a representative (but not exhaustive) group of leaders in the field to build a consensus on the best practices for describing the methods utilized in fNIRS studies.

Our paper has been designed to provide guidelines to help enhance the reliability, repeatability, and traceability of reported fNIRS studies and encourage best practices throughout the community. A checklist is provided to guide authors in the preparation of their manuscripts and to assist reviewers when evaluating fNIRS papers.

## 1 Motivation

Functional near-infrared spectroscopy (fNIRS) is a noninvasive, easy-to-use, and portable brain imaging technology that enables studies of normal brain function and alterations that arise in disease, both in the laboratory as well as in real-world settings.^1^^–^^4^ In 1977, Jöbsis used the technique for the first time to noninvasively assess changes in human brain oxygenation due to hyperventilation.^5^ Since then, the tool has evolved into an established noninvasive brain imaging modality and has been applied to a wide range of different populations and research questions.

The volume of fNIRS research has dramatically increased over the last two decades^1^ in parallel with the growing availability of commercial fNIRS systems. This rapid growth has resulted in a great diversity in methodological practices, data processing methods, and statistical analyses.^6^ While the diversification of research methods is expected and welcomed in such a fast-growing field, it can present challenges in the interpretation, comparison, and replication of different fNIRS studies. The lack of standardized pipelines in the analysis of neuroimaging data and the resulting differences in study results is not unique to fNIRS, with concerns also being raised by the Functional Magnetic Resonance Imaging (fMRI) community.^7^ This problem is exacerbated by poor reporting practices that can considerably hinder or bias the review process and dramatically reduce a given paper’s impact and subsequent replicability. The purpose of this paper is to offer researchers guidelines on how to report fNIRS studies in a comprehensive, transparent, and accessible way. These guidelines are not intended as standards; rather, they are best practices on how to report an fNIRS study to ensure the full impact of the findings is achieved.

This paper follows the structure of a typical fNIRS research paper and each section (Introduction, Methods, etc.) discusses the guidelines relevant to that section. We provide a comprehensive checklist in the Appendix (Table 1) with references to the relevant sections in order to facilitate revisiting of the text for more details. It is worth noting that, for the sake of brevity, instrument-related guidelines presented here focus on continuous wave NIRS (CW-NIRS) technology and only briefly refer to the other existing NIRS technologies [frequency-domain NIRS (FD-NIRS), time-domain NIRS (TD-NIRS), and diffuse correlation spectroscopy (DCS)].

## 2 Title, Abstract, and Introduction

### 2.1 Good Title and Abstract Structure

#### 2.1.1 Choosing a good title

A good title is critical for a scientific paper. It should be both informative and specific, short and concise, and contain sufficient information about the content and topic of the paper.^8^ As was shown by Paiva et al.,^9^ scientific papers have higher citations and viewing rates when the title is short, does not include a question mark, a colon, or a hyphen, and is a “results-describing title” rather than a “methods-describing title.” For example, having the paper title “Using functional near-infrared neuroimaging to study the neuronal correlates of language development in children from age 2 to 14: A new study” might well be replaced by “Language development causes age-dependent changes in cerebral activation in Broca’s area.”

#### 2.1.2 Structured abstract: Clarity and consistency

Abstracts are highly compressed versions of a paper that deliver its core findings and significance. The presentation and scientific quality of an abstract are generally good predictors of these qualities in the rest of the paper. A good abstract is “informative” and “motivating.” The quality of an abstract is correlated with the number of times the paper is cited^10^ and guides the initial decision in the publication process.^11^

We recommend implicitly or explicitly structuring the abstract similar to the main body of the paper, i.e., “Introduction,” “Aims,” “Methods,” “Results,” and “Conclusion,” addressing some or all of these, as appropriate, in a few sentences, unless the journal itself requires a different abstract structure. The Introduction part provides the objective of the study backed up by the necessary scientific background and motivated by its significance to the field. The Aims part itemizes the objectives of the study. In the Methods part, the most relevant aspects of the methodology should be concisely reported such as the experimental design/stimuli, if relevant, the sample size, brain regions of interest, major data processing, and/or statistical analysis steps. Papers that introduce a new methodological advancement in fNIRS hardware or data processing should provide key details such as the validation of the new method. The Results part reports the main outcomes of the paper including the most relevant numerical results, such as hemodynamic change in regions of interest and their statistical significance. The data or results included in the abstract should also be reported in the main body of the manuscript and match with the data/results therein. The Conclusion part synthesizes the interpretation of the results at hand and their possible significance/impact in the field.

### 2.2 Introduction Sections in fNIRS Papers: Structure and Content

#### 2.2.1 Scope, context, significance, and aim of the work

As for all research papers, the introduction of an fNIRS research paper serves to convey the scope, context, innovation, and significance of the study being reported. It typically (1) outlines the general research question of the study, (2) reviews the literature that is relevant to the central research question of the study, highlighting existing knowledge and knowledge gaps, (3) motivates the reported study, (4) describes the specific hypotheses and/or predictions being tested, (5) provides a brief summary of the methods that will be used to test the hypotheses, and (6) states the specific aims of the current study. Given that fNIRS now is a known methodology, it is not necessary to always reference basic validation papers. The Introduction section for technological and methodological papers should describe how and why the innovative technique/method differs from existing ones, what advantages are expected, and how the method has been validated. For papers dealing with clinical, neurological, or neurocognitive questions, the Introduction section should focus on how the research question makes an advance in our understanding of brain function, brain disease, or neurocognitive mechanisms. Moreover, if relevant, the rationale for using fNIRS over other neuroimaging modalities should be elucidated. A clear and succinct statement of the aims of the study at the end of the introduction section helps the reader build appropriate expectations. These aims should correspond with the conclusions drawn at the end of the paper.

## 3 Methods: Making a Study Reproducible

The methods section should enable the reader to understand how the results were achieved and how to reproduce the results. It should contain information on the participant demographics, details of the experimental paradigm, the system used, data acquisition details, and the preprocessing steps including the statistical methods used. The section should also include a figure showing (1) the measurement set up (a high-quality original photograph from a measurement session or a drawing), (2) the fNIRS optode array/channel configuration on the head, (3) a visualization of the experimental protocol, and optionally (4) a sensitivity analysis to show how well the fNIRS set up is able to probe the regions-of-interest chosen for the study.^12^^–^^14^ Moreover, if the signal processing pipeline is complex and involves advanced and/or innovative steps, it is highly recommended to include a block diagram that depicts all the processing steps along with input and output signals. It is worth noting that some journals have the method section at the end as an appendix. In these cases, the introduction and result sections should provide sufficient methodological information to understand the context without the need to go into methodological details.

### 3.1 Participants

#### 3.1.1 Human participants

The sample of participants is typically described with a set of the most relevant demographics and, if appropriate, clinical characteristics. These include the number of participants, their mean age and variation, or age range with a precision that is most useful (e.g., hours for newborns, months and days for infants), and the gender distribution. The inclusion and exclusion criteria should be clearly defined (e.g., pathologies, native language, etc.). Other relevant features, such as handedness, ethnicity, socio-economic status, etc., may also be provided. It is worth noting that it may be relevant to report the ethnicity distribution, especially if it is different from what may be expected from the population at the location where the study was conducted. fNIRS signal quality can be dependent, among other things, on hair properties (color, thickness, and density). A biased selection of participants may result in the lack of generalizability of the fNIRS neuroimaging findings. For multiple group studies, the procedure for group assignment should be described.

For clinical populations, the amount of disease-related information depends on the focus of the paper. Depending on the study (e.g., clinical populations), it may be advisable to briefly provide key characteristics in the manuscript and refer to a (supplementary) table for epidemiological details. Typically, a table would list the time since onset, the cause of the brain lesion/dysfunction (e.g., ischemic cardiogenic left middle cerebral artery stroke), and relevant clinical findings (e.g., residual aphasia). For specific populations, if applicable and available, it may be useful to report biomarkers, such as blood markers (e.g., anemia, which can lead to altered or unexpected results^15^^,^^16^), parameters related to the overall physiological fitness or the specific pathology assessed. If data from some participants were not included in the final analysis, then the demographics of the final sample should also be provided along with the data rejection criteria. To ensure transparency and safeguard against biased rejection, it is also important to specify at what point during data processing the different rejection criteria were applied and whether they were applied in batch or on a case-by-case basis. Information regarding ethical issues must be provided including the name of the institutional review board (IRB) that assessed and approved the study protocol, the ethical procedures followed (e.g., obtaining informed consent, minor assent, and/or parental permission) as well as a link to the clinical study registration, if available.

#### 3.1.2 Sample size and statistical power analysis

An appropriate sample size, or number of participants, is important for any fNIRS experiments, but there is no fixed rule to guarantee statistical validity. One practical approach to determine the sample size is to perform a power analysis, which estimates the minimum sample size needed to obtain a certain effect size at a preset power level (1−β) (which is 1 − probability of a type II error, conventionally set to 0.8) and α (the probability of a type I error, conventionally set to 0.05).^17^ A power analysis report typically contains the sample size (the necessary sample size for an *a priori* power analysis and the actual sample size for an *a posteriori* power analysis), the power (selected power for an *a priori* power analysis and achieved power for an *a posteriori* power analysis), and alpha levels utilized, the effect size chosen along with its justification (e.g., prior research or pilot study), the relevant statistical tests for hypothesis testing, and relevant citations for the platform used to perform the power analysis.

### 3.2 Experimental Paradigm and Instructions

#### 3.2.1 Experimental design (or “study design”)

Some specifics of the fNIRS signal must be considered when designing the experimental paradigm. For example, the dominance of physiological confounds in fNIRS signals^18^ (see Sec. 3.5), which means that each stimulation condition must almost always be repeated multiple times to allow the functional response to be resolved. Meanwhile, the temporal characteristics of the hemodynamic response place limits on the duration of the interval between consecutive stimuli if the data are to be block averaged. Physiological confounds that are temporally correlated with the stimulus also need to be considered. For instance, a participant’s breathing pattern may align with the stimulation blocks if they are presented at regular intervals. This may increase false-positive responses.^19^ These issues can be minimized through thoughtful experimental design that reduces anticipatory effects, for example, by pseudo-randomizing both the order of conditions and the length of the interstimulus interval. These considerations may be informative to report when describing the experimental design of the study.

An accurate description of the experimental design is critical both to the reader’s understanding of the results of an fNIRS study and to the reproducibility of the work. Any feature of an experiment that could feasibly affect the results or their interpretation should be reported in the methods section. Wherever possible it is recommended to include a schematic of the experimental paradigm.

The vast majority of fNIRS paradigms fall into one of the following categories: Block design, event-related design, and resting-state paradigms for functional connectivity studies. In the case of resting-state paradigms that do not include explicit stimulation of the participant(s), the paradigm can be aptly described by the details of the duration of recording; the environment in which the participant is placed (e.g., lighting conditions, auditory conditions, eyes open/closed, objects or displays in their visual field, etc.); and by any instructions given to the participant (see Sec. 3.2.2).

**Fig. 1:**
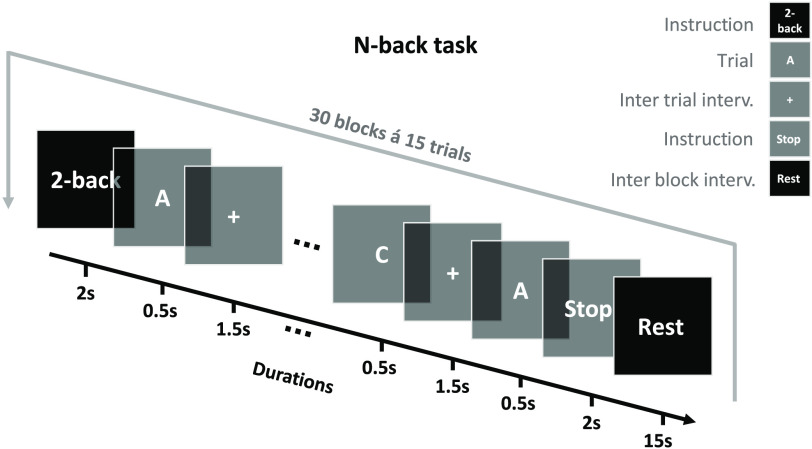
Experimental paradigm visualization. Sample legend follows. Schematic illustration of the n-back paradigm. Each experimental run consisted of 30 blocks with an interblock interval of 15 s. Each block has 15 trials and starts with the task instruction “n-back” displayed for 2 s on the screen. After the instruction, letters are displayed on the screen, one at a time, for 0.5 s. The intertrial interval is 1.5 s, during which a fixation cross is displayed on the screen. Participants were instructed to indicate whether the current letter is identical to the one presented “n” trials preceding it.

The features that should additionally be reported for both block- and event-related paradigms include: The stimuli, the number of conditions, the number of blocks or trials per condition, the order in which the blocks or trials are presented, the duration of each block or trial, and the duration of interblock or intertrial intervals. A sketch providing timing and examples of the stimuli (e.g., still images depicting frames of a visual stimulus) can be highly informative. Figure 1 shows an example.

#### 3.2.2 Participant instructions, training, and interactions

fNIRS papers should provide a clear description of what instructions about the task were given to the participants. Instructions can often be crucial for the interpretation of the neural data. For instance, explicit instructions about learning a stimulus set versus implicit exposure to the same stimulus set may trigger different attentional, motivational, and learning mechanisms. Therefore, aspects relevant to how participants conceive of and complete the task need to be mentioned, e.g., time constraints on responses, explicit or implicit task, description about the objective of the task, etc. Similarly, feedback given to the participants or other incentives that may change their attention or motivation to perform the task need to be explained. Experimental conditions that may have influenced the participant’s performance during data acquisition, such as overly long set up procedures, acquisition under dim/dark lighting conditions, environmental distractions, etc., also need to be reported.

### 3.3 System and Acquisition

#### 3.3.1 fNIRS device and acquisition parameters description

The fNIRS research field has been rapidly expanding both in technological innovations and neuroscience applications, leading to the development of a variety of commercially available and custom in-house developed devices.^2^^–^^4^^,^^20^^,^^21^ Instruments differ not only in their fundamental mode of hardware operation, but also in the methodological procedures applied to recover chromophores oxy- and deoxyhemoglobin (thus also total hemoglobin), and/or cytochrome-c-oxidase^22^ concentration changes or optical signals reflecting them (abbreviated by ${\mathrm{HbO}}_{2}$, Hb, tHb, and CCO, respectively). [It is worth noting that other acronyms (e.g., HbO/HbR/HbT, $O_{2}\mathrm{Hb}/\mathrm{HHb}/\mathrm{tHb}$, or oxy-Hb/deoxy-Hb/total Hb) are also common and acceptable.]. Therefore, accurate reporting of the fundamental aspects of the instrument specifications is mandatory. While most commercial fNIRS instruments are CW, they do not necessarily use the same near-infrared (NIR) wavelengths or the same algorithms for recovery of the hemoglobin concentrations. In addition, a significant number of custom-built fNIRS instruments tend to implement technologies such as TD-NIRS,^23^ FD-NIRS,^24^ or high-density (HD) technology,^25^ which have fundamental differences from current commercial instruments; a fact that is mostly unknown to the nonexpert user. Accurate reporting of relevant instrument specifications will allow for better interpretation of the research study and a higher level of transparency for replication. The publication should clearly report the following information when describing fNIRS device specifications: (1) manufacturer and version, (2) mode of operation (CW, FD, and TD), (3) number and spectrum of wavelengths, (4) irradiance (source power over area of exposure) or average power or both [care should be taken that the light source exposure complies with the safety standards such as ANSI (United States) or IEC-60825 (Europe)], (5) sampling rate, number and type of optodes and resulting channels, and source–detector distances, and (6) method for the data conversion to chromophore concentration (if automatically done by the instrument’s software; otherwise this will be reported in the data analysis section). The information can be given in a brief summarizing sentence, such as “We used an NIRSdev (NIRScomp, country) CW-NIRS device with 24 active channels (8 laser diode emitters, $\lambda_{1|2}=750|850\;\mathrm{nm}$ with average power <1  mW, and 8 avalanche photodiode detectors) sampled at 50 Hz. Data were converted to concentration changes using the modified Beer–Lambert law (mBLL).” All assumptions (fixed scattering and water concentration) and parameters for the conversion [such as extinction coefficients and differential pathlength factors (DPF)] should be reported, including how changes in DPF are accounted for, e.g., in longitudinal studies of infant development. References for the chosen parameters may also be reported. If FD or TD devices were used, the procedures employed to obtain absorption and scattering coefficients should be stated. More guidance on the use and reporting of mBLL parameters and units is provided in relation to data analysis in Sec. 3.4.3.

#### 3.3.2 Optode array design, cap, and targeted brain regions

The reproducibility of fNIRS measurements strongly depends on clear documentation of the design (geometry) and placement of the source–detector array. Although fNIRS technologies are rapidly evolving, most fNIRS studies still feature a limited field of view and/or channel density, thus the layout of sources and detectors on the scalp vary from study to study. Many fNIRS devices are equipped with sets of sources and detectors or “optodes” that can be arranged flexibly. Others come with predefined pads of sources and detectors, or fixed distributions that can be freely positioned, but not reorganized. Determining an appropriate position and arrangement of optodes for a given fNIRS study is, therefore, a necessity.^26^^,^^27^ However, this process is far from trivial as fNIRS measurements are highly dependent on the position, extent, source–detector separation(s), and density of the fNIRS source and detector array.^28^^,^^29^ These factors affect the sensitivity of the measurement to a given cortical region, the relative contributions of the brain and extracerebral tissues to each signal, and the homogeneity of the measurement sensitivity across the field of view. Digital head models (virtual phantoms/simulations) can be used to understand device-specific NIR light propagation, vital for designing next-generation optical brain imaging devices and optode arrays. Monte Carlo simulations^30^^,^^31^ provide a controlled mechanism to characterize and evaluate contributions of diverse fNIRS sensor configurations and parameters such as optical path length, detector surface area, and source–detector separation.^32^^–^^34^

**Fig. 2:**
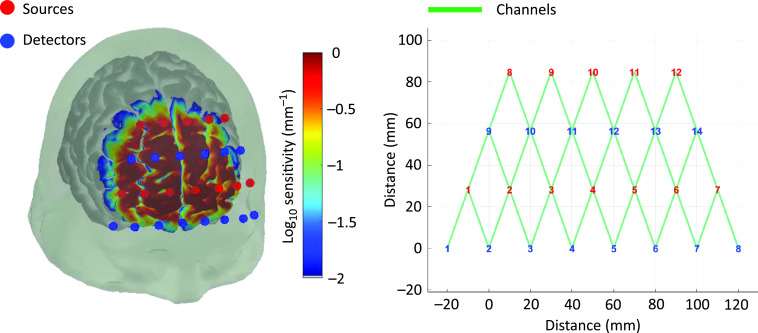
Example of optode array set up with 12/14 source/detectors resulting in 34 channels over prefrontal cortex with 30-mm separation. Sensitivity profile in $\log_{10}({\mathrm{mm}}^{-1})$. Visualization using AtlasViewer.^14^

When reporting an array design in a publication, we strongly recommend including a diagram of the array that specifies: (1) the total number of source and detector positions; (2) the total number of channels; and (3) the distribution of source–detector separations. It is also beneficial to include a photograph, where possible, of the array in position on a participant. This may provide additional information regarding the physical design and ergonomics of the array. Figure 2 shows an example.

The process of placement and registration of the array to the head of the participants should also be accurately described to facilitate reproducibility across participants and across studies. Source and detector locations (or a subset thereof) should be described in relation to cranial landmarks such as the nasion, inion, ears (e.g., the preauricular points), and/or electroencephalography (EEG) 10-20, 10-10, and 10-5 landmark points. This can be noted directly (e.g., “Source 1 was placed at 10-20 position C3.”) or relatively (e.g., “Source 1 was placed on the midline 3 cm above the nasion.”).

It is also highly recommended to report the location of the fNIRS array and the associated channel sensitivity distributions relative to the underlying cortical macroanatomy. These anatomical locations can then be reported in terms of gyral labels (e.g., inferior frontal gyrus), Brodmann areas (e.g., BA44), Montreal Neurological Institute coordinate space, Talairach coordinate space,^35^ or via the inclusion of figures depicting the cortical sensitivity map associated with the array.^36^

A description of how these anatomical locations were determined should also be provided. For example, the simplest registration approach is to position the array relative to certain 10-5 coordinates and directly determine the underlying anatomy via the 10-5 coordinates of an atlas model.^12^ However, this assumes the array positioning is identical across participants, and that the atlas provides an accurate model of the cohort. Alternatively, participant-specific registration of the fNIRS array can be performed using information derived from three-dimensional (3D) positioning systems, neuro-navigation technologies, or via photogrammetry approaches.^26^^,^^37^^,^^38^ In this case, researchers can additionally report the variance in the optode locations on the scalp and/or the variance in underlying macroanatomy. Any instrument, software, or processing approach used to achieve spatial registration and what assumptions those approaches rely upon should be described. If an atlas is used, the source of the atlas should be provided, and the limitations associated with the use of that atlas should be acknowledged.

#### 3.3.3 For publications on instrumentation/hardware development

As progress in new fNIRS designs and innovations continues across the globe,^39^ specific guidelines and standardizations are needed to streamline the efforts and accelerate the adoption of the new technologies. These efforts can be facilitated by first disseminating the use of standard naming conventions in device specifications (see sample nomenclature in Table 2). While for older devices, a reference of a paper describing the device may be sufficient, if the focus of the paper is to present new technology, a description of the new device should include (1) a hardware block diagram, depicting connections and control mechanism, (2) software flowchart, describing flow of information and the control of hardware components and data acquisition protocol, (3) the type of light source and detectors, (4) the measures taken to prevent external contamination and cross-talk across channels (such as time-multiplexing, frequency multiplexing, or a combination of both), and if possible, (5) circuit diagrams of key components and individual part numbers. If digital head models are used to guide the hardware design, they should be properly cited.

The type of light source (laser/LED), specific wavelengths, and the emitted power per unit area (e.g., $0.2\;W/{\mathrm{cm}}^{2}$) need to be reported to assess safety level and potential classification of the device. NIR light exposure to eye and skin (if needed, exposure after protective gear) should remain within universally accepted safety norms, such as the International Standard for Safety of Laser Products^40^ or the International Standard for Photobiological Safety of Lamps and Lamp Systems.^41^

The type of the light detector (e.g., pin photodiode, avalanche photodiode, photomultiplier tube, single-photon avalanche detector, etc.), its configuration (e.g., single pixel photodiode, photodiode array, imaging charge-coupled device, etc.), its light sensitivity profile for specific wavelengths of interest (gain, noise factors, and noise equivalent power), and skin interface style (direct contact, use of light-guides or fibers) should be noted.

For developers and manufacturers of fNIRS instrumentation, especially for regulatory approval, it is essential to be aware of the recently published International Electrotechnical Commission (IEC)/International Organization for Standardization (ISO) standard for fNIRS equipment (IEC 80601-2-71), a particular standard in the 60601 family of standards for medical electrical equipment.^42^ As in any electrical instrument, product safety testing should be certified independently (e.g., Underwriters Laboratories-UL marking in the United States, Consumer Electronics-CE marking in EU, Product Safety Electrical Appliance and Materials-PSE in Japan, and China Compulsory Certificate-CCC mark in China). For university grown systems, this could be done via local hospital biomedical engineering departments that test the electrical safety of these research devices before use with humans. For university researchers, use of new optical brain imaging devices in clinical/research studies only requires local ethics committee approval. For eventual clinical deployment such as diagnostics or therapeutics, further regulatory approvals are required (e.g., FDA in United States, EU MDR in Europe, Pharmaceutical and Medical Device Act-PMDA in Japan, and National Medical Products Administration-NMPA, formerly CFDA, in China).

To achieve comparability and reliability in clinical studies, standardized performance assessment of fNIRS instrumentation based on dedicated phantoms should be an important part of instrumentation development. The aforementioned IEC 80601-2-71 standard also includes several performance tests on turbid phantoms. The main test relies on an fNIRS phantom with a realistic overall attenuation and a changeable internal aperture to create a defined attenuation change that corresponds to a certain change in ${\mathrm{HbO}}_{2}$ and Hb. Other phantom-based tests described in this standard include signal stability, response time, signal-to-noise ratio (SNR), and signal cross-talk.

A more comprehensive performance characterization and comparison of diffuse optics instruments and methods is facilitated by several protocols based on multilaboratory consensus-building efforts [e.g., Optical Methods for Medical Diagnosis and Monitoring of Diseases (MEDPHOT) protocol, Basic Instrumental Performance (BIP) protocol, and Noninvasive Imaging of Brain Function and Disease by Pulsed Near Infrared Light (nEUROPt) protocol].^43^^–^^45^ The nEUROPt protocol^45^ specifically targets fNIRS instrumentation, aiming at characterizing contrast, contrast-to-noise ratio (CNR), lateral resolution, depth sensitivity, and quantification of absorption changes in the brain. It is implemented by homogeneous turbid phantoms with small black inclusions, e.g., a solid–solid switchable phantom^46^ and by two-layered phantoms. Other fNIRS phantoms have been reported mimicking the temporal change of ${\mathrm{HbO}}_{2}$ and Hb concentrations, e.g., by means of electrochromic variable absorbers^47^ or movable layers.^48^^,^^49^ Hb-containing phantoms with variable oxygenation for tissue oximeter testing^50^ should also enable quantitative assessment of fNIRS signals. Creating anatomically realistic dynamic phantoms can be challenging, but is possible.^51^^–^^53^

Papers describing instrumentation development should report the following data for the specific phantom tests that were performed: phantom type, its optical and geometrical parameters, the test arrangement including source–detector separation(s), and results of the test(s). For an example, see Ref. 54.

Although commercially available fNIRS devices seldom come with an accompanying phantom, developers and manufacturers of fNIRS instrumentation could benefit from the adoption of established guidelines for phantom-based tests^55^ for routine quality checks. An overall check of reproducibility of signal magnitude is useful to identify problems such as fiber breaking and degradation of light sources or detectors. If phantom-based routine tests are recommended by the manufacturer, the procedures adopted for the preparation and characterization of the phantom should be reported.

### 3.4 Preprocessing Steps

**Fig. 3:**
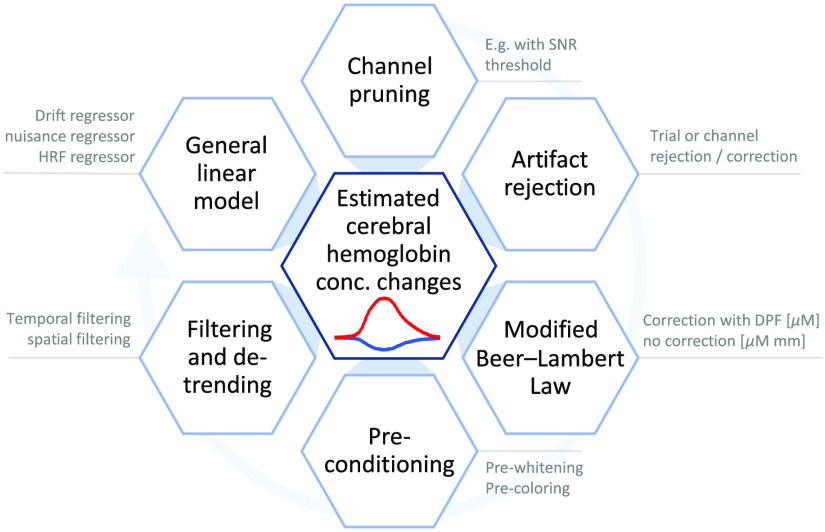
Overview of elemental fNIRS preprocessing steps. Light blue circular arrow indicates conventional processing order. It is worth noting note that, depending on the analysis, not all steps are always present or necessary.

To facilitate the reproduction of scientific findings and to ensure that important processing steps are not skipped during analysis, the methods section should include a detailed description of all the data analysis steps. Figure 3 summarizes the main preprocessing steps in an fNIRS data analysis pipeline and the following sections present the expected level of detail with which they should be presented in the methods section.

#### 3.4.1 fNIRS signal quality metrics and channel rejection

An important preprocessing step in fNIRS data analysis is the signal quality check of the raw signal for each channel. The noise in fNIRS signal may originate either from the measurement system (e.g., due to light source instability, electronic noise, and shot noise), which we call merely “noise,” or of physiological origin or head/body motion which we call “confounding signals” throughout the paper.

The fNIRS signal quality check on noise can be tested either by a simple SNR check or by obtaining cardiac power at each channel using spectral analysis. When the sampling rate is reasonably high (e.g., 10 Hz), the heartbeat is a good indicator of optode-scalp coupling and thus a good quality control metric for the fNIRS signal. The Methods section may thus include an indication of the SNR threshold (e.g., >20  dB) and cardiac power threshold^56^ utilized to reject data channels from further analysis. As it is likely that different measurement channels will fail the criteria for different participants, one should also report the number of participants remaining for each channel to avoid any misinterpretation of the results.

Especially in fNIRS, due to the various types of confounding signals and noise, it is important to be aware of the conceptual differences between the metrics SNR, CNR, and contrast-to-background ratio (CBR) and to use these terms unambiguously. The term SNR should be used to quantify the signal quality of an instrument’s fNIRS channel. It is calculated from the measured raw light intensity within a fixed time window and is expressed as $\mathrm{SNR}=20\;\log_{10}(\frac{\mu }{\sigma })$, where μ corresponds to the signal’s intensity offset (dc component) and σ corresponds to the signal’s variance (ac component). Contrast metrics (CBR/CNR) are used when the strength of an extracted hemodynamic response is to be related to background confounding signals or measurement noise and thus depend on the specific preprocessing of the signal. For more details on these metrics, refer to Ref. 57.

#### 3.4.2 Motion artifacts

The fNIRS signal may contain motion artifacts in the form of spikes or baseline shifts, especially in data collected from noncompliant populations, such as infants (see Sec. 3.6.7) or during experimental tasks that require motion (walking or speaking). In such cases, either the motion artifacts can be identified and adjacent trials can be removed from analysis or one of the many motion artifact correction algorithms in the literature can be used.^58^ In either case, handling and correction of motion artifacts and related parameters should be reported (e.g., the thresholds for identifying motion, the specific parameters of the correction method). Moreover, as the former method (i.e., identifying and removing trials that overlap with motion artifacts) will lead to a reduction of the number of trials within a run, the number of remaining trials should be reported. Finally, the output of the motion artifact removal algorithm needs to be verified via theoretical and empirical methods for assessing the performance of the algorithms (see an example on how to verify a new algorithm^59^).

#### 3.4.3 Modified Beer–Lambert law, parameters and corrections

Changes in optical densities or absorbance are converted into changes in hemoglobin species ${\mathrm{HbO}}_{2}$ and Hb by applying the mBLL.^60^ In CW-NIRS, the mean pathlength traveled by the detected photons, however, is not known. In a highly scattering medium, the pathlength of trajectories is longer than the source–detector separation. One can estimate the pathlength within the whole sampling region by multiplying the source–detector distance with a DPF that was experimentally obtained with FD-NIRS or TD-NIRS.^61^^–^^64^ Thus, when reporting, one option is to use a DPF (taken from the literature) and report the results in changes in chromophore concentration in molar concentration units, e.g., μM. This option takes into account the wavelength and source–detector distance dependence of the pathlengths and is, therefore, more appropriate when comparing information from channels of different separations. When DPF data are not available, researchers may rely on another option, which is not to use a mean pathlength to extract concentration changes from the Beer–Lambert law.^65^ In this case, the signal changes are presented as the products of concentration changes and mean pathlength, in units of (molar concentration × distance), e.g., μM cm or μM mm. The latter approach may be appropriate when a single separation is used, but has limitations for multiple separations.

It is worth noting that the changes in chromophore concentration may vary dramatically depending on the processing method and whether a correction is applied, and if so, which correction method is applied. As an example, for the same measurement channel, the resultant ${\mathrm{HbO}}_{2}$ concentration change can be reported as: 40  μM mm without any correction and 0.22  μM when a pathlength correction is applied with a differential pathlength correction factor of 6 and a source–detector distance of 30 mm. In all cases, the method of choice and relevant parameters (e.g., DPF) should be stated and citations should be provided. The units should be clearly labeled when presenting the concentration changes results.

#### 3.4.4 Impact of confounding systemic signals on fNIRS

The NIR light traveling from source to detector interrogates the cerebral cortex, but to a larger extent also the extracerebral tissue layers. Changes in blood flow and oxygenation in the extracerebral tissues (in particular in the scalp) affect the fNIRS signals and result in potential misinterpretation of the signals measured.^4^^,^^19^ In addition, systemic physiological changes also affect cerebral hemodynamics. The main sources of physiological confounds are (1) changes in partial pressure of ${\mathrm{CO}}_{2}$ (${\mathrm{PaCO}}_{2}$),^66^ systemic blood pressure,^67^ changes in heart rate and vascular tone both in the extracerebral as well as the cerebral tissues due to the interplay between the autonomic nervous system and the sympathetic nervous system^68^ and (2) changes in blood flow and oxygenation due to head movements, teeth clenching, or eyebrow raising.^69^^–^^71^

Neglecting physiological confounding effects may result in both false positives, i.e., wrongly assigning a detected hemodynamic change to functional brain activity, or false negatives, i.e., masking brain activity when it is present.^19^^,^^72^ Therefore, it is recommended to employ a systemic physiology augmented fNIRS approach, where these systemic parameters are measured simultaneously.^73^ On the other hand, recognizing and isolating these changes in systemic physiology provides innovative insights into the complex regulation of brain hemodynamics involving, for example, networks that react particularly to neuronal activity or to systemic physiological changes.^74^ Most of the effort in fNIRS (pre-) processing focuses on separating or rejecting confounding signals and there are various strategies that can be employed, the most prominent being the general linear model (GLM). This topic is discussed again in more detail in Sec. 3.5.

#### 3.4.5 Strategy for statistical tests and removal of confounding signals

The aim of an fNIRS study typically falls into one of these categories for statistical testing: (1) comparison of brain responses to task versus baseline, (2) comparison of brain responses during different tasks, and (3) correlations between hemodynamic signals within a brain or across brains. These test results are highly affected by the particular noise structure of the fNIRS data. Noise in fNIRS data is frequency-dependent (colored) and correlated, due to strong physiological components (cardiac, respiration, and variations in blood pressure). As these features violate the main assumption in the GLM that the noise is not frequency-dependent (white) and is uncorrelated,^75^ it is necessary before employing a GLM analysis to either (1) prefilter the data to remove confounding signals such as physiological confounds and motion artifacts, and/or to (2) prewhiten the signal,^76^^,^^77^ and/or to (3) precolor the signal.^78^^,^^79^ As an example of prewhitening methods^77^ intrinsic temporal correlation of fNIRS data can be estimated using autoregressive models. Inversion of the temporal correlation estimates is then employed in generalized least squares to obtain unbiased and efficient estimates of GLM parameters. On the other hand, this inversion in the prewhitening method is sensitive to the correct estimation of temporal correlation. Therefore, as an alternative method, one can use the temporal filter (smoothing) matrix to estimate the temporal correlation of fNIRS data. This precoloring method is valid when the low-pass filter with sufficiently large kernel width is applied to fNIRS data. Then, least squares can be applied to the temporally smoothed data with the GLM extended to include the filter matrix.^80^ This process yields unbiased parameter estimates, but does not retain their maximal efficiency. In all cases, the method chosen and the prefiltering steps should be clearly stated.

#### 3.4.6 Filtering and drift regression

High-frequency components in the signal such as instrument noise and cardiac pulsations are often removed using a low-pass filter (e.g., Butterworth filter or Chebyshev filter). The low-pass filter threshold, if too low, can also remove the brain response of interest and thus should be chosen carefully (typically 0.5 Hz or higher). On the other hand, much lower frequency components in the signal can be removed using a high-pass filter. Using a value too high as a threshold can remove the actual desired brain signal, especially if the duration of the experimental task block is comparable to the high-pass threshold (e.g., 0.05-Hz high-pass filter versus 20 s of stimulus duration). The type of filtering applied, the order of the filter, if any, and the cut-off frequency should be stated (e.g., a third-order zero-phase Butterworth bandpass filter with cutoff frequencies of 0.01 to 0.5 Hz). It is critical to understand the phase response, as filters with nonlinear phase response would distort the signal. Finite impulse response (FIR) filters have linear phase responses and can be applied safely both offline and online (during data collection) unlike infinite impulse response (IIR) filters (e.g., Butterworth), which require zero-phase correction and can only be applied offline as the correction requires the entire signal at once. An alternative approach to filtering is to add a drift factor into the GLM as a regressor to model the low-frequency oscillations in the data (e.g., third-order polynomial drift). The respiration and Mayer wave oscillations, on the other hand, fall into the same frequency range as the hemodynamic response and cannot simply be removed by bandpass filtering.^81^

### 3.5 Physiological Confounds in the fNIRS Signal: Strategies

#### 3.5.1 Strategies for enhancing the reliability of brain activity measurements

**Fig. 4:**
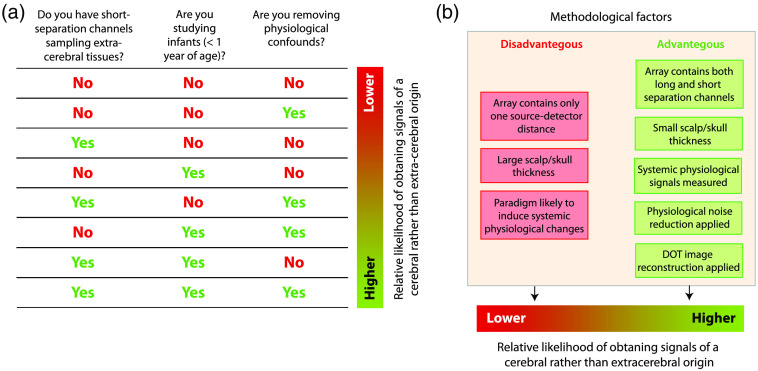
The likelihood of measuring real hemodynamic changes in the cerebral cortex is determined by the depth-sensitivity of the fNIRS measurements and the impact of confounding systemic physiological signals. Checklist for estimating the likelihood of obtaining cerebral signals (a), methodological factors that affect the likelihood of obtaining signals of cerebral origin (b).

Due to the presence of physiological confounds in fNIRS signals, it is not recommended to report results based on signals measured only with long-separation channels and without dedicated signal processing which takes into account possible confounding systemic physiological changes, particularly in adult participants with thicker overlying extracerebral tissues. The relationship between the fNIRS instrumentation (with respect to the source–detector arrangements used) and the likelihood of measuring real hemodynamic changes in the brain (cerebral cortex) is illustrated in Fig. 4. This chart applies, in particular, when CW-NIRS devices are employed. The likelihood of detecting brain-activity related changes is high when (1) significant changes in systemic physiology can be excluded, (2) a depth-sensitive multidistance fNIRS approach is used and the data are processed in such a way that the interference from changes in the extra-cerebral layer is filtered, or (3) a CW-NIRS set up is used with only long source–detector separations for each channel, but specific signal-processing is applied to the signals to reduce the confounding influence of the extracerebral tissues. In the absence of short-separation channel measurements, a large number of channels or additional measurements of systemic physiology can help in cleaning the signal. The following sections summarize established approaches for these strategies.

#### 3.5.2 Strategy 1: Enhance depth sensitivity through instrumentation and signal processing

This approach requires the extension of the fNIRS measurement set up so that the measurements are depth sensitive, i.e., being able to differentiate between changes in the extracerebral and cerebral layers. To achieve depth sensitivity, the fNIRS set up should contain optical channels with source–detector separations of different lengths and with short ones in particular (Fig. 4). A short-separation channel (<15  mm, optimum distance ∼8  mm for adults and ∼4 to 5 mm for infants^82^) is mostly sensitive to blood perfusion and oxygenation changes in the extracerebral tissue layer. With CW-NIRS, the parallel usage of short- and long-separation channels maximizes sensitivity to the cerebral cortex while minimizing the sensitivity to the extracerebral layers. Such measurements made with short-separation channels enable to regress out the signal changes in the extracerebral layer from the long-separation channel, an approach commonly termed “short-separation regression” and pioneered by Saager and Berger.^83^ Several methods have been developed to perform the regression, including least-squares algorithms and diverse types of adaptive or Kalman filtering.^4^ A recent promising development is the innovative combination of the GLM approach with temporally embedded canonical correlation analysis for the analysis of fNIRS data.^84^ Previous work proved that short-separation regression decreases the trial-to-trial variability of the hemodynamic response^85^ and reduces the impact of strong hemodynamic changes happening in the extracerebral layer.^86^

Other depth-sensitive instrumentations involve: (1) multidistance measurements, which use diffusion theory and the signal slope from multiple source–detector separations,^87^^–^^90^ (2) diffuse optical tomography (DOT) {other acronyms [e.g., diffuse optical spectroscopy (DOS), near-infrared imaging (NIRI), diffuse optical imaging (DOI), near-infrared optical tomography (NIROT), and high-density tomography (HD-tomography)] are also acceptable}, which provides depth-resolved measurements using a very large number of channels,^91^^–^^95^ and (3) TD-NIRS systems, which measure the time of flight of photons: The depth is encoded in the arrival time of the photons since late photons have traveled deeper.^23^^,^^96^

#### 3.5.3 Strategy 2: Signal processing without intrinsic depth sensitive measurements

In the case of a subideal measurement (i.e., only long-separation channels available), one should strive to decompose the data into brain activity and physiological confounds (Fig. 4). One approach is to approximate systemic changes with the global component from the mean (or median) of all channels and to filter it from each channel.^97^ Alternative approaches are data-driven signal processing methods that decompose the fNIRS signals into its brain and systemic components (blind source separation methods, e.g., independent component analysis and principal component analysis).^4^

#### 3.5.4 Strategy 3: Incorporating measurements of changes in systemic physiology in the fNIRS signal processing

When additional systemic physiological signals are available (e.g., heart rate, respiration rate, respiration volume, arterial ${\mathrm{CO}}_{2}$ concentration, blood pressure, and skin conductance), they enable to (1) regress out these influence from the fNIRS signals and/or (2) investigate in detail the relationships of these signals with the fNIRS signals. This can be done, for example, with a GLM approach that uses the systemic physiological signals or linear time-lagged mixtures of these signals as additional regressors.^84^ The details of the processing and generation of such regressors should be reported (e.g., the signals included and the phase/time lag used). It is worth noting that both strategies 2 and 3 have the risk of removing brain activity or failing to properly remove systemic physiological components due to the heterogeneity of the vasculature within the scalp.^98^

### 3.6 Analysis and Statistical Methods

#### 3.6.1 Hemodynamic response function estimation: Block averaging versus general linear model

Calculating hemoglobin concentration changes using the mBLL is generally followed by the estimation of hemodynamic response function (HRF) by simple block averaging, convolution, or linear estimation models. The GLM represents measured data as a linear combination of functionally distinct components. While block averaging avoids *a priori* assumptions about the shape of the HRF, the GLM allows modeling different confounding factors in the fNIRS signal along with the hemodynamic response to the stimulus. The GLM enables simultaneous estimation of the contribution of the fNIRS components and thus provides a less biased estimate of the HRF. GLM reports should include all regressors modeled along with their parameters as well as the method used to estimate the weight of the regressors (e.g., “The HRF was modeled using Gaussian functions with a standard deviation of 0.5 s and their means separated by 0.5 s. The weights of the regressors were obtained using an ordinary least squares fit.”). The report should also include the number of trials included in the final analysis, if the total number is reduced from the number reported in the experimental protocol due to various reasons (e.g., motion artifact contamination).

#### 3.6.2 HRF estimation: Selection of the HRF regressor in GLM approaches

The HRF is typically modeled either by a fixed canonical shape (e.g., a gamma function variant) or by more flexible models such as a linear combination of multiple basis functions, e.g., Gaussians. To increase the statistical power, fixed canonical shapes are advantageous provided that the shape of the HRF is known *a priori*.^99^^,^^100^ The methods of the paper should include the model and its parameters as well as a justification for the model preference in cases where a fixed shape is chosen. However, if the shape of the HRF is not known (in different populations, experimental paradigms, brain regions, etc.), using a fixed model can result in a loss in statistical power and bias the results. In such cases, flexible models are preferred as they allow capturing the true temporal characteristics of the HRF.

#### 3.6.3 Statistical analysis: General remarks

Claims formulated in a paper should be supported by statistical analysis. All statistical analyses are linked to the experimental design and the underlying hypotheses, and thus, there is no single standardized way of describing the statistical analysis. If part of this information is missing, the accuracy of the statistical methods cannot be verified and results cannot be compared across studies or future replications. Reporting effect size and confidence intervals is strongly recommended, as each is a sample-size-free statistic, and thus enables a more convenient comparison across different studies. Using tables and figures to present statistical results improves readability.

#### 3.6.4 Statistical analysis of GLM results

The weights of the GLM regressors at each channel are typically estimated using a least squares method that minimizes the sum of the squared differences between the actual and fitted values. In terms of the type of least square methods, ordinary least squares is based on the model assumption that errors are uncorrelated between observations. Therefore, when there is a degree of temporal correlation between the residuals in a regression model, one can use a generalized least squares approach either with prewhitening or with precoloring.^80^ Statistical inference is then performed by testing the null hypothesis, i.e., that estimated coefficients are not significantly different from zero. Rejection of the null hypothesis indicates that there is a response to the stimulus. Generally, hypothesis testing of single contrasts (i.e., a linear combination of effects) is executed using a t-statistic, whereas multiple contrasts are simultaneously tested using an F-statistic. Therefore, when reporting the GLM analysis results, it is important to describe which regressors were included in the contrast and to address the specific statistical tests applied. In the second-level GLM analysis, population effects can be estimated using fixed-effects, random-effects, or mixed-effects analysis.^101^^,^^102^ In contrast to the fixed-effects, the random-effects models take into account both sources of variation (within-subject and between-subject variability), and thus allows making inferences about the population from which the sample is drawn. In either case, it is essential that authors clearly describe the method used for the second-level analysis in their paper. Finally, statistical significance of channel-specific effects is assessed by thresholding a test statistic Z (e.g., t- or F-statistic) at a height z. Multichannel fNIRS systems come with the cost of a high risk of type I error (false positive) due to the large number of concurrent statistical tests for each channel, thus, type I error control is essential. This so-called “multiple comparisons problem” will be discussed in the following section.

#### 3.6.5 Statistical analysis: Multiple comparisons problem

When a single channel or region of interest is analyzed based on *a priori* knowledge, statistical inference can be made based on an uncorrected p-value. However, if statistical analysis is performed on multiple channels, regions, or network components, a statistical inference should be adjusted to reduce the risk of the type I error (false positive) by correcting for multiple comparisons. Multiple comparisons should be corrected or controlled by appropriate methods including Bonferroni correction,^103^ Holm correction, false discovery rate control,^104^ effective multiplicity correction, random field theory, or permutation tests.^79^^,^^94^^,^^102^^,^^104^^,^^105^ Random field theory is suitable for interpolated fNIRS topographic maps. An appropriate method should be selected for relevant statistical inference with the research purpose. Authors are encouraged to clearly describe their specific approach of correction and report p-values labeled according to the type of correction. Also, when cluster-based inference is used, the threshold for the cluster size should be reported with an adjusted p-value.

#### 3.6.6 Specific guidelines for data processing in clinical populations

While processing steps are largely identical to other populations, pathology leads to some caveats. Since clinical studies often aim to detect a sign of pathology in the individual rather than at the group level, demonstrating differences between neurotypical controls and a cohort of patients may uncover a pathology related trait, but often does not allow for diagnostic or therapeutic guidance. The potentially complex interaction between changes in behavior (i.e., the effect of the neurological deficit) and a disease-related alteration in brain function is another challenge to be dealt with in the analysis. Alterations in brain function may relate to neuronal signaling (e.g., epilepsy), to the vascular response (e.g., stroke/ cerebrovascular disease), and an alteration of neurovascular coupling (e.g., in dementia^106^^,^^107^). Moreover, pathology may alter optical properties of the sampled tissue including changes in the thickness of different layers [e.g., atrophy increases cerebrospinal fluid (CSF) space] or in their absorption and scattering properties (e.g., blood in CSF due to subarachnoid hemorrhage^108^).

Other considerations regarding data processing in clinical populations are as follows. (1) Variability in behavior: Lesser performance may result in lesser activation irrespective of pathology. Conversely, recruitment of additional brain areas to achieve near-normal task performance is potentially an indicator of brain pathology. It is, therefore, advisable to include performance/behavior into the analysis and/or report it in the publication. Since typical fNIRS approaches sample from a quite limited part of the brain surface, performance should be coregistered with precision. This allows for factoring out or correlating fNIRS data with metrics of task performance, offering a way to disentangle general and task-specific aspects of the fNIRS results. (2) Integration of clinical data: In addition to disease severity, site of the lesion, comorbidity, and premorbid performance range all contribute to variability across clinical participants and should, therefore, be reported. If clinical data are available, it is highly advisable to integrate these data into the analysis. (3) Integration of coregistered data: In clinical populations, conflicting results from large arrays of data in different modalities (e.g., fNIRS/EEG data) are often interpreted to signal pathological alteration. It should be kept in mind, however, that methodologies differ with regard to the areas or physiological signals sampled, as well as the response dynamics, and that this is convolved with the impact of pathology. Reference data from nonaffected brain areas within the same participants may increase sensitivity and should thus be reported whenever appropriate. The reliability of the results is enhanced if responses in a pathological brain area or functional system are compared to a reference system, which is shown to be unaffected.

#### 3.6.7 Specific guidelines for data processing in neurodevelopmental studies

Analysis and testing of data from developmental populations is largely identical to that of adults. However, data are often of smaller quantity and/or noisier in quality. A lack of understanding or compliance with instructions, lower motor control, and a shorter attention span in infants and young children lead to fewer numbers of trials in a study and/or larger number of motion-related artifacts.^109^ In adults, corrupted data segments are often corrected or replaced using central tendencies of the surrounding data or the entire dataset (e.g., by interpolation), which, to work, requires sufficient volume and quality. In developmental data, this method may not always work. Nevertheless, correction methods should also be used with developmental data,^110^ provided that the data used for correction are of sufficient quality. Alternatively, data rejection may be used. The rejection procedure needs to be well documented in the manuscript to avoid biasing the results (details of the rejection criteria, the amount of data rejected, whether rejection was manual or automatic, etc., need to be reported). The higher noise and artifact levels of developmental data may also increase variability and reduce statistical significance. Despite these challenges, fNIRS data acquisition and analysis are quite successful in infants, since infants’ smaller head sizes and thinner skulls and tissues allow for a deeper penetration and better visibility into the cortex. Age-appropriate experimental designs and adequate attention getters can also prevent some of the motion artifacts and attentional limitations.

#### 3.6.8 Connectivity analysis

Functional connectivity is defined by the temporal correlations between time courses of hemodynamic changes of two distinct brain regions.^111^ Using signals measured at two fNIRS channels, the relationship between two regions can be evaluated by calculating Spearman’s correlation, the lagged correlation, mutual information, entropy, the phase locking index, wavelet transform coherence, and so forth, typically in a low-frequency range (e.g., 0.009 to 0.10 Hz).^112^ While reporting a connectivity analysis, one should include the calculation method, the frequency band of interest, the preprocessing methods^113^ applied in the analysis, whether the correlation analysis was performed on the raw signal or ${\mathrm{HbO}}_{2}/\mathrm{Hb}$ time series, and whether it was intrahemispheric or interhemispheric, or if region of interest (ROI)-based, whether it was within ROI or between ROIs. A sensitivity analysis showing how the results change when selecting a different frequency band is also helpful and provides additional insights into the underlying physiology of the connectivity measures. For dynamic resting-state functional connectivity analysis, one should also report the duration of the time window and step size (e.g., “A Pearson correlation coefficient was calculated between any two measurement channels using a sliding window correlation approach with a time window of 100 s and a step size of 5 s.”). Transformations before statistical testing (e.g., Fisher z-transform), the statistical thresholds, and the method of correction for multiple comparisons should also be reported.^114^

Although functional connectivity determined by fNIRS signals measured from appropriate source–detector distances mostly reflects cerebral hemodynamic changes rather than superficial contamination for infants,^115^ this may not be the case for adults. This is quite critical as the symmetrical vasculature anatomy on scalp may strongly contribute to the resultant high correlations in long-separation channels. While partial correlations among multiple channels may reduce the effects of superficial and global signals,^116^ the most reliable approach is to use a depth-resolved instrumentation such as DOT or TD-NIRS. The paper should report the specific procedure for dealing with physiological confounds from scalp and how this issue can significantly bias the results. The discussion should also include the fact that fNIRS does not measure signal changes in deep cortical regions, thus the interpretation of the results is always limited to the measured cortical surfaces. It is important to keep in mind that two close fNIRS channels might also reflect some spurious connectivity just because they are partly sensitive to the same underlying cortical brain region through the fNIRS forward model.

#### 3.6.9 Image reconstruction

DOT provides a mapping from source–detector measurements y, on the head surface, to local hemodynamic changes within the head volume x via a differential model A called the sensitivity matrix (or the Jacobian) by solving the linear equation y=Ax. Image reconstruction provides greater anatomical specificity of the optical data, facilitates anatomy referenced subject averaging, and within-/cross-group- and cross-modal comparisons. Reports should include sufficient details on methods, software, and parameter selection for each of the following five major steps in the pipeline for fNIRS-based image reconstruction. (1) The head anatomy is ideally provided by participant-specific anatomical magnetic resonance imaging (MRI) volume,^117^^–^^119^ though atlas-based approaches can also work quite well when the atlas is a good match for registration to the participants.^120^^–^^123^ The model has three essential pieces of information: The size and shape of the head, the internal distribution of optical properties, and the location of the optical array elements on the surface. (2) The selected head anatomy is segmented into a set of putative tissues. These parameters as well as the segmentation method need to be reported. (3) Head mesh generation: for any model, the number of labeled tissue regions and their optical properties should be reported. (4) The optical array is localized on the meshed anatomy via methods such as electromagnetic localization^37^^,^^120^^,^^124^ or referencing to EEG-standards such as the 10-20.^125^ Accurate coregistration of the optical elements to their true location on the head surface is essential as mismatches lead directly to spurious results pointing to inappropriate brain areas. (5) Once the array is localized on the tissue, sensitivity profiles (A) of the source–detector measurements are generated by modeling the light transport in tissue using Monte Carlo simulations (e.g., using TOAST++^126^ or MCX^127^) or the diffusion approximation (e.g., using NIRFAST^128^). The model of choice needs to be stated. (6) The sensitivity matrix, A, is then inverted using appropriate regularization (e.g., Tikhonov, spatially variant, total variation, or elastic net). Multiple software suites exist that support image reconstruction pipelines (e.g., NeuroDOT,^129^ AtlasViewer,^12^ NIRS-SPM,^49^ and NIRSTORM^13^) with direct tunable interaction for optimization and processing. All the applied processes/methods must be clearly documented to enable unambiguous reproduction of the results.

#### 3.6.10 Single trial analysis and machine learning

Domains such as brain–computer interfaces, neuroergonomics, and neurofeedback focus on single-trial and/or real-time decoding of fNIRS signals and increasingly incorporate machine learning. Machine learning may provide powerful tools for analysis and classification of brain signals, but requires the user to consciously avoid common mistakes and to document how good practice in data science was ensured.

Classification methods exploit any discriminable evoked changes and artifacts in the signal. Consequently, non-neuronal signal components induced by emotional or physical activity, such as scalp blood flow, may lead to false positives and improved discriminability in the experiment, but dramatically reduce the decoding performance outside of the constrained paradigm. This pitfall re-emphasizes the importance of appropriate separation of confounding signals and brain signals, as discussed in Sec. 3.5. Efforts to classify brain signals only and to interpret the classifier weights physiologically should be clearly reported.

The strict separation of the analyzed data into training and test sets is crucial to avoid overfitting and reporting flawed performance results. It is important to ensure that any statistical inference from the data during learning must be limited to the training set. This does not only include model selection/training of classifiers, but also data-based channel or feature selection or the training of regressors or filters for processing. If the dataset is too small to split it into separate training and testing partitions, cross-validation schemes can be applied. If automatic selection of additional parameters, e.g., fNIRS feature selection, is to be performed, the cross-validation should be nested. If a regressor is learned, for instance, the across-trial HRF shape using a GLM approach, the GLM needs to be embedded in the cross validation.^130^ Applying a learned filter or the GLM on the entire dataset before single trial analysis invalidates the integrity of the approach. All steps for the training and selection of models and parameters should be reported to allow methodological assessment and reproducibility.

#### 3.6.11 Multimodal fNIRS integration

With the perspective of measuring complementary physiological parameters ${\mathrm{HbO}}_{2}$ and Hb, the integration of fNIRS in multimodal studies is becoming more frequent. Historically, first simultaneous fNIRS/fMRI studies^131^^–^^133^ aimed to clarify commonalities and improve quantification of hemoglobin during activation. Quantification was also studied combining either fNIRS or TD-NIRS with Positron Emission Tomography (PET).^134^^,^^135^ Early integration of fNIRS with EEG^136^^–^^138^ and Magnetoencephalography (MEG)^139^ aimed to investigate neuro-vascular coupling processes. These early studies helped to develop specific guidelines to report multimodal studies.

A strong rationale is required to justify the practical difficulties as well as the cost of multimodal studies. Therefore, any multimodal fNIRS study should first describe the motivations to combine fNIRS with another modality, which are usually falling within one of the four main categories listed below: (1) providing improved quantification of brain hemodynamics and oxygenation (e.g., combining hemoglobin measurements with fMRI, DCS, or CCO measurements to provide quantitative measurements of physiologically interpretable parameters such as ${\mathrm{CMRO}}_{2}$ and hemoglobin^22^^,^^140^^–^^145^), (2) assessing brain activity at the time of complex or transient events, usually monitored and detected using scalp EEG^146^ (e.g., prolonged recordings to characterize hemodynamic responses to epileptic discharges,^136^^–^^138^^,^^147^^–^^149^ sleep physiology^150^^,^^151^ and sleep disorders,^152^ or resting-state fluctuations^153^), (3) monitoring brain activity in real time for brain–computer interfaces^154^ and during noninvasive brain stimulation,^155^ or (4) when experimental designs involving complex cognitive processes can benefit from simultaneous recordings to better explore the underlying complex neural processes (e.g., language, learning, attention, intention, emotion).^156^^–^^158^

When reporting fNIRS multimodal studies, the set up of the acquisition should be carefully described, especially the methods considered to synchronize the different modalities in time. To fully benefit from the added value of multimodal approaches, accurate sensor localization and coregistration are also helpful,^35^ notably through the use of neuro-navigation tools. We, therefore, recommend to include a detailed figure of the experimental set up. Other issues to be considered are (1) guiding optical fibers in the scanner for fNIRS/fMRI corecording and ensuring optode-scalp coupling, (2) simultaneous montage arrangement with EEG (integrated fNIRS/EEG sensors,^159^ optimal montage design integrating fNIRS and EEG positions,^148^^,^^160^ and gluing optical fibers on the scalp^148^^,^^161^ are among the options), and (3) fNIRS sensor profile and thickness for simultaneous fNIRS/Transcranial Magnetic Stimulation (TMS), fNIRS/MEG, and fNIRS/fMRI acquisitions (e.g., low-profile sensors have been considered to keep the TMS stimulation coil close to the scalp^155^).

Each multimodal approach requires a unique method to combine the data and simultaneously analyze it. For instance, in simultaneous fNIRS/EEG, EEG oscillations or transient discharges can be used to model the fNIRS response using GLM-based approaches. Integration of tomography, statistical methods, and brain normalization can facilitate future studies and one should promote the development of software packages allowing the analysis of several functional modalities (fMRI, fNIRS, and EEG/MEG) within the same environment (e.g., NIRS-SPM^79^ and NIRSTORM^13^).

## 4 Results: How and What to Report

### 4.1 Figures and Visualization

Good visualizations that depict all relevant information in a clear and presentable way enable readers to understand complex information quickly and easily. The Results section should include visualizations of both chromophores ${\mathrm{HbO}}_{2}$ and Hb and statistical outcomes (e.g., t-values) on a brain/head template, or a justification if one of the two chromophores is not reported. When reporting statistics, the rules set by the American Psychological Association should be followed^162^ {e.g., “There was a significant increase in ${\mathrm{HbO}}_{2}$ signal during the task period [mean±SD: 20±5  μM mm; one sample t-test, t(23)=2.5, p<0.05, Cohen’s d=0.5].”}. Average ${\mathrm{HbO}}_{2}$ and Hb time-series for each channel or a selected set of channels or ROIs at the subject or group level are of great benefit for providing the temporal characteristics of the change as well as data quality.^19^ In such plots, providing standard deviations is a minimum requirement. To illustrate the statistical contrasts, it is often useful to also show the data as box plots or distributions that include single data points.^163^^,^^164^ If the analysis is focused on prediction/classification using machine learning, established data science reporting should be followed. Among the tools used for visualizing statistics and performance are receiver operating characteristic plots, confusion matrices, and scatter plots showing statistical distributions.

A strategy that has proven itself to create high-quality figures is to first create the raw figures using the signal processing and data analysis tools used (e.g., MATLAB^®^, R, Python) and then to finish the images with a professional vector graphics software.

#### 4.2 Concise Text and Rigor

The results section should be very concise and well organized, presenting only, but completely, the results obtained with the methods described. If the journal has length restrictions, some of the results can be shown in the supplemental material. Results that have been published previously should be clearly delimited from new results. A bias toward publication of results that confirm the tested hypothesis is often observed, possibly harming objectivity.^165^ It is, therefore, highly recommended to report all analyses undertaken, irrespective of whether the results are positive or null. Also, it is good practice to separate planned analyses, decided upon prior to data analysis (if these were preregistered at an open science platform, then a URL to the preregistered study plan can be provided, see Sec. 7.1), and exploratory analyses, inspired by the data during analysis. Highlighting null results or results that contradict the original hypotheses are important for transparency and replicability.

## 5 Discussion and Conclusion: The Implications of the Work for the Bigger Picture

### 5.1 Discussion of the Results in Light of Existing Studies: Strengths, Limitations, and Future Work

In the Discussion, previous findings in the same or related fields (fNIRS, fMRI, EEG, or other) should be compared and contextualized with the existing results. This ensures a consistency check with the literature and brings out the innovative contributions and significance of the findings. Correlation and causality should not be confused, and causality should not be reported without evidence.

Discussion should ideally have a separate section dedicated to the strengths and limitations of the study. The strengths of an fNIRS study could include an innovative experimental paradigm employed, an in-depth study of a particular neural/cognitive phenomenon, a large sample size, or the development and application of an innovative hardware or signal processing method. Limitations could be small sample size, instrumentation, and presence of confounding effects in the measurements and analysis. For instance, although fNIRS brain sensitivity is higher in younger populations due to smaller scalp/skull thickness,^82^ a study, if performed without an independent measurement of the extracerebral hemodynamic changes (e.g., via short-separation channels) should still consider physiological confounders in their analysis and discuss possible implications of physiological noise on their results and interpretation.

A dedicated description of potential next steps of research based on the work presented in the manuscript enables the discussion of open scientific questions and ideally includes the formulation of hypotheses that the new work generated, which can be investigated in the follow-up work.

#### 5.2 Conclusion

The Conclusion should synthesize the main findings of the study and summarize its significance and impact for the field in a very concise form. The conclusion needs to be consistent with the aims and with the results. It is recommended that the conclusions are carefully considered and defined first. This helps writing a consistent straightforward publication.

## 6 Bibliography

### 6.1 Proper Citations

Familiarity with the literature in the area of research is a prerequisite for the contextualization of the presented work. To provide context and a rationale, and to compare the findings with the existing literature, it is essential to include relevant review articles and original research articles on the specific topic at hand. Consequently, in the final draft, each reference should be double-checked to verify that the information referred to in the manuscript is in agreement with the one presented in the cited original work.

## 7 Supplementary Data: Reinforcing Reproducibility

### 7.1 Preregistration, Data, and Code Sharing

Study and analysis plans can be preregistered before data acquisition begins. Such practice ensures transparency and allows researchers to distinguish between planned and exploratory analyses and interpret their findings accordingly. Studies can be preregistered on a number of open science repositories such as Neuroimaging Tools and Resources Collaboratory (NITRC), GitHub, rOpenSci, Dryad, Open Science Framework (OSF), Mendeley, Figshare, and arXiv. Many of these also allow data and code sharing once the study is completed. Sharing data and code with the research community facilitates the reproducibility of the findings as it allows researchers to independently test and verify the results, and to obtain new discoveries and interpretations without the unnecessary repetition of the work. Consequently, we strongly encourage the sharing of fNIRS data and code, as well as other useful information such as stimuli presented during the experiment. Some journals provide the opportunity to share this additional information as a supplementary to the main body of the paper. If such options do not exist, one of many other avenues might be used such as online repositories. A link to the relevant repository can be provided in the methods section of the paper. One advantage of using such resources is that they allow logging downloaders who have access to the data, as is required by many ethics’ committees. When data from human studies are openly shared, it is crucial to ensure that it has been completely de-identified. IRBs are great resources to get guidance on the protection of human privacy while sharing data. Openly sharing hardware/software is also quite useful and can further speed up innovative technological developments (e.g., the opennirs^166^^,^^167^/openfnirs^168^^,^^169^ projects). Finally, data should be shared in an openly and broadly accessible format. The fNIRS community is adapting a common fNIRS data format: The “shared near-infrared data format,” or “snirf” (https://github.com/fNIRS/snirf). Using a common standard format and standard guidelines such as compatibility with the Brain Imaging Data Structure,^170^ already adopted by most other neuroimaging modalities, can greatly facilitate data sharing across research groups that use different acquisition systems and processing pipelines.

## 8 Appendix

Table 1 is a checklist for guiding authors in the preparation of their manuscripts, and Table 2 is a list of commonly used fNIRS nomenclature.

**Table 1:** The following checklist is provided as a means to summarize the guidelines in this article to help the reader cross-check whether s/he can further improve the manuscript before submission. Each question refers to a numbered section in the main text that can be consulted again for more detail.

Topic	Checklist
2.1.1 Choosing a good title	Is the title short, specific, and informative about the results?
2.1.2 Structured abstract: Clarity and consistency	Is the most relevant information described in a motivating way? Can you reduce the abstract further to improve clarity? Is the abstract structured similarly to the structure of the main body of the paper? Is the data in the abstract and main manuscript consistent and complete?
2.2.1 Scope, context, significance, and aim of the work.	Is the scope, context, and significance of the work established? Has the previous work been described and cited properly? Are the aim and hypothesis clearly defined?
3.1.1 Human participants	Are all relevant demographic, clinical, and other relevant characteristics described? Are all participant and data inclusion/exclusion criteria clearly defined? Are all ethical issues and procedures discussed? Is approval from the local ethics committee clearly addressed? Are excluded participants disclosed and well justified?
3.1.2 Sample size and statistical power analysis	In cases where no effect is observed: Was a power analysis performed? Was the selection of sample size, power, alpha levels, and effect size reported and justified? A posthoc power analysis may state the sample size needed to achieve statistical significance in case the study was underpowered.
3.2.1 Experimental design (or “study design”)	Is the following information provided for the study design? All studies: The duration of recording; the environment in which the participant is placed (e.g., lighting conditions, auditory conditions, objects or displays in their visual field, etc.). Specific to block- and event-related designs: The number of conditions; the number of blocks or trials per condition; the order in which the blocks or trials are presented; the duration of each block or trial; and the duration of interblock or intertrial intervals. A diagram that provides details of the timings of stimulus and images of the stimuli themselves.
3.2.2 Participant instructions, training, and interactions	Were incentives, instructions, and feedback to the participants clearly outlined? What experimental conditions could have influenced the participant’s performance?
3.3.1 fNIRS device and acquisition parameters description	Is the acquisition set up and instrumentation sufficiently described? (system, wavelengths, sample rate, number of channels, and other parameters)
3.3.2 Optode array design, cap, and targeted brain regions	Is the description of optode array design, cap, and targeted brain regions complete?
3.3.3 For publications on instrumentation/hardware development	Are all crucial hardware and software performance characteristics and validation steps reported? Are the architecture and all crucial components (light source, detector, and multiplexing strategies) sufficiently described? What standards/norms were followed and what safety regulations were considered (i.e., maximum permissible skin exposure)? For instrumentation or methods development papers: Is phantom-based performance characterization reported? For application studies: Are regular system quality checks reported?
3.4.1 fNIRS signal quality metrics and channel rejection	How was signal quality of fNIRS channels checked and were bad channels rejected?
3.4.2 Motion artifacts	How were motion artifacts identified and removed?
3.4.3 Modified Beer–Lambert law, parameters and corrections	What were the assumptions, parameters, and models selected to derive concentrations from the raw fNIRS signals using the mBLL? How were estimation errors corrected/what are the signals’ units?
3.4.4 Impact of confounding systemic signals on fNIRS	How did your study distinguish between the variety of physiological processes that comprise fNIRS signal changes? Have you considered all factors of possible physiological confounds?
3.4.5 Strategy for statistical tests and removal of confounding signals	Have the overall preprocessing and statistical testing strategies clearly been identified and outlined?
3.4.6 Filtering and drift regression	How were confounding signals outside of the main fNIRS band of interest tackled? (High/low-pass filtering/GLM drift regression)
3.5.1 Strategies for enhancing the reliability of brain activity measurements	What strategies were pursued to correct for physiological confounds and changes in the extracerebral tissue compartment? How were confounding signals identified and separated and what was done to reduce the likelihood of false positives/negatives?
3.5.2 Strategy 1: Enhance depth sensitivity through instrumentation and signal processing	How was depth sensitivity achieved? If multidistance measurements were performed, what are the source–detector separations used? What signal processing methods were applied to remove confounding physiological components in the fNIRS signals? How are the limitations discussed?
3.5.3 Strategy 2: Signal processing without intrinsic depth-sensitive measurements	If no depth-sensitivity/multidistance measurements are available: What signal processing methods were applied to minimize confounding physiological components? How are the limitations discussed?
3.5.4 Strategy 3: Incorporating measurements of changes in systemic physiology in the fNIRS signal processing	If other physiological signals were used for the removal of confounding signals in the fNIRS signals, which ones? Are all relevant parameters and steps sufficiently described?
3.6.1 Hemodynamic response function estimation: Block averaging versus general linear model	What is the effective number of trials used for HRF estimation? In GLM approaches: What confounding signal regressors were used and how were they modeled? What method was used to estimate regressor weights?
3.6.2 HRF estimation: Selection of the HRF regressor in GLM approaches	In GLM approaches: How was the HRF modeled? What shape/function was used for the HRF regression? What are the parameters? If a fixed shape was used, what is the justification?
3.6.3 Statistical analysis: General remarks	What statistical tests were performed and are all corresponding parameters (e.g., assumed distribution, degrees of freedom, p-values, etc.) reported? Is the effect size stated?
3.6.4 Statistical analysis of GLM results	What regressors were included in GLM to explain effects of interest and confounds for fNIRS data? What statistical model and methods have been used for testing the hypothesis at the first and second levels?
3.6.5 Statistical analysis: Multiple comparisons problem	If statistical analysis was performed on multiple regions/voxels/network components, were family-wise errors corrected? What correction method was applied?
3.6.6 Specific guidelines for data processing in clinical populations	Are clinical variability and expected alterations of behavioral, neuronal, and vascular responses considered when interpreting the results?
3.6.7 Specific guidelines for data processing in neurodevelopmental studies	How were the increased noise, artifacts, and analysis handled specifically for the developmental populations? Is the artifact rejection procedure well documented in the manuscript?
3.6.8 Connectivity analysis	What correlation indices have been used? How were the statistical thresholds determined?
3.6.9 Image reconstruction	What head anatomy was used and how was coregistration between optical elements and head geometry performed? How was the head anatomy segmented and into what tissue types? How was the head mesh generated? What optical properties were used for each tissue type? What model/approach was used for the generation of sensitivity profiles and image reconstruction?
3.6.10 Single trial analysis and machine learning	What efforts were undertaken to understand and interpret the classifier weights and outputs? What was the training and test size, how were (hyper-) parameters selected? Was training and test data strictly separated, especially in approaches that use learned filters, regressors, or the GLM? Was cross validation performed and if yes, what kind?
3.6.11 Multimodal fNIRS integration	Was the sensor coplacement/localization/registration sufficiently described? What were the methods used for data fusion and multimodal analysis?
4.1 Figures and visualization	Was the measurement set up, optode array configuration and placement, and experimental protocol visualized? Is a sensitivity analysis included? If the processing pipeline is complex, is it depicted in a simplified block diagram? Are both brain maps and time courses available and provided? Are results linked to anatomical locations? Are both ${\mathrm{HbO}}_{2}$ and Hb reported? Are higher order statistics of the data visualized as well?
4.2 Concise text and rigor	Are the results presented in a concise and well-organized manner? What efforts were undertaken to minimize confirmation bias? Are negative results reported, if present?
5.1 Discussion of the results in light of existing studies: Strengths, limitations, and future work	Are all relevant results discussed? Is any part of the discussion based on results that were not presented? Were caveats from confounding physiology sufficiently addressed? Is the presented work sufficiently compared and contextualized with existing studies? Are strengths and weaknesses clearly outlined and discussed? Are potential next steps discussed?
5.2 Conclusion	Are 3 to 5 conclusions drawn that summarize the main findings of the study in a concise way? Do they include the significance of the result? Are the conclusions based on the results of the study?
6.1 Proper citations	Are all the statements that reference to an original work agree with the information provided therein?
7.1 Preregistration, data, and code sharing	Is data/code made available to other researchers to reproduce the results? Is data shared in a common data format that the community supports (e.g., snirf)?

**Table 2:** Useful nomenclature.

Channel	Unique/independent measurement area that the system is capable of recording.
	Note: Any time series originating from the same optode, such as different wavelengths or oxygenated/deoxygenated hemoglobin, still belongs to the same channel measurement.
DPF	Scaling factor that relates geometrical source–detector distance to the average pathlength light travels between the source and detector within the entire sampling region, accounts for the increased distance that light travels from the source to the detector due to scattering.
Frame	One concurrent/corresponding sample from all channels.
Frame rate	Rate at which frames were recorded in Hz.
Frequency multiplexing	Distinguishing different channels by modulating the sources at nonoverlapping frequencies.
Mean pathlength	The pathlength light travels within the entire sampling region (source–detector distance multiplied by DPF).
Partial pathlength	The path light travels within the fraction of tissue that is of interest, e.g., for functional brain activation, this is the path only in the activated region (source–detector distance multiplied by partial pathlength factor).
Partial pathlength factor	The scaling factor that relates source–detector distance to the average pathlength light travels within the activated region.
Partial volume effect	Underestimation of the concentration changes due to the fact that changes in hemoglobin occur in a focal region rather than in the entire sampling region.
Partial volume error	Error that occurs when the partial volume effect is different between the different wavelengths which may lead to inverse traces.
Sampling rate	Number of samples collected per second (in Hz) from each channel.
Time multiplexing	Distinguishing different channels by turning them on one at a time or in groups.
