# Errata: Best practices for fNIRS publications

**DOI:** 10.1117/1.NPh.8.1.019802

**Published:** 2021-02-08

**Authors:** Meryem A. Yücel, Alexander v. Lühmann, Felix Scholkmann, Judit Gervain, Ippeita Dan, Hasan Ayaz, David Boas, Robert J. Cooper, Joseph Culver, Clare E. Elwell, Adam Eggebrecht, Maria A. Franceschini, Christophe Grova, Fumitaka Homae, Frédéric Lesage, Hellmuth Obrig, Ilias Tachtsidis, Sungho Tak, Yunjie Tong, Alessandro Torricelli, Heidrun Wabnitz, Martin Wolf

**Affiliations:** a Boston University, Neurophotonics Center, Biomedical Engineering, Boston, Massachusetts, United States; b Massachusetts General Hospital, Harvard Medical School, MGH/HST Athinoula A. Martinos Center for Biomedical Imaging, Department of Radiology, Charlestown, Massachusetts, United States; c University Hospital Zurich, University of Zurich, Department of Neonatology, Biomedical Optics Research Laboratory, Neonatology Research, Zurich, Switzerland; d University of Bern, Institute for Complementary and Integrative Medicine, Bern, Switzerland; e Université de Paris, CNRS, Integrative Neuroscience and Cognition Center, Paris, France; f Università di Padova, Department of Social and Developmental Psychology, Padua, Italy; g Chuo University, Faculty of Science and Engineering, Applied Cognitive Neuroscience Laboratory, Tokyo, Japan; h Drexel University, School of Biomedical Engineering, Science and Health Systems, Philadelphia, Pennsylvania, United States; i Drexel University, College of Arts and Sciences, Department of Psychology, Philadelphia, Pennsylvania, United States; j Drexel University, Drexel Solutions Institute, Philadelphia, Pennsylvania, United States; k University of Pennsylvania, Department of Family and Community Health, Philadelphia, Pennsylvania, United States; l Children’s Hospital of Philadelphia, Center for Injury Research and Prevention, Philadelphia, Pennsylvania, United States; m University College London, DOT-HUB, Department of Medical Physics and Biomedical Engineering, Biomedical Optics Research Laboratory, London, United Kingdom; n Washington University School of Medicine, Department of Radiology, St. Louis, Missouri, United States; o University College London, Department of Medical Physics and Biomedical Engineering, London, United Kingdom; p Washington University School of Medicine, Mallinckrodt Institute of Radiology, St. Louis, Missouri, United States; q Concordia University, Department of Physics and PERFORM Centre, Multimodal Functional Imaging Lab, Montreal, Québec, Canada; r McGill University, Biomedical Engineering Department, Multimodal Functional Imaging Lab, Montreal, Québec, Canada; s Tokyo Metropolitan University, Department of Language Sciences, Tokyo, Japan; t Polytechnique Montréal, Department Electrical Engineering, Montreal, Canada; u University Hospital Leipzig, Max Planck Institute for Human Cognitive and Brain Sciences and Clinic for Cognitive Neurology, Leipzig, Germany; v Korea Basic Science Institute, Research Center for Bioconvergence Analysis, Ochang, Cheongju, Republic of Korea; w Weldon School of Biomedical Engineering Purdue University, West Lafayette, Indiana, United States; x Politecnico di Milano, Dipartimento di Fisica, Milan, Italy; y Consiglio Nazionale delle Ricerche, Istituto di Fotonica e Nanotecnologie, Milan, Italy; z Physikalisch-Technische Bundesanstalt, Berlin, Germany

## Abstract

Errors in the Acknowledgments section of the originally published article are corrected.

This article [*Neurophotonics* **8**(1), 012101 (2020) doi: 10.1117/1.NPh.8.1.012101] was originally published on 7 January 2021 with erroneous attributions in the Acknowledgments section.

Original:

“fNIRS; F.S., Strategy for statistical tests and removal of confounding signals; M.Y and S.T., Filtering and drift regression;”

Corrected:

“fNIRS; M.Y. and S.T., Strategy for statistical tests and removal of confounding signals; M.Y, Filtering and drift regression;”

Original:

“M.Y., fNIRS signal quality metrics and channel rejection”

Corrected:

“M.Y. and A.L., fNIRS signal quality metrics and channel rejection”

The article was corrected and republished on 13 January 2021.

